# Regulating fatty acids in infant formula: critical assessment of U.S. policies and practices

**DOI:** 10.1186/1746-4358-9-2

**Published:** 2014-01-16

**Authors:** George Kent

**Affiliations:** 1 Department of Political Science, University of Hawai’i, Honolulu, HI 96822, USA

**Keywords:** Infant formula, Fatty acids, Regulation, Docosahexaenoic acid, DHA

## Abstract

**Background:**

Fatty acids in breast-milk such as docosahexaenoic acid and arachidonic acid, commonly known as DHA and ARA, contribute to the healthy development of children in various ways. However, the manufactured versions that are added to infant formula might not have the same health benefits as those in breast-milk. There is evidence that the manufactured additives might cause harm to infants’ health, and they might lead to unwarranted increases in the cost of infant formula.

The addition of such fatty acids to infant formula needs to be regulated. In the U.S., the Food and Drug Administration has primary responsibility for regulating the composition of infant formula. The central purpose of this study is to assess the FDA’s efforts with regard to the regulation of fatty acids in infant formula.

**Methods:**

This study is based on critical analysis of policies and practices described in publicly available documents of the FDA, the manufacturers of fatty acids, and other relevant organizations. The broad framework for this work was set out by the author in his book on *Regulating Infant Formula*, published in 2011.

**Results:**

The FDA does not assess the safety or the health impacts of fatty acid additives to infant formula before they are marketed, and there is no systematic assessment after marketing is underway. Rather than making its own independent assessments, the FDA accepts the manufacturers’ claims regarding their products’ safety and effectiveness.

**Conclusions:**

The FDA is not adequately regulating the use of fatty acid additives to infant formula. This results in exposure of infants to potential risks. Adverse reactions are already on record. Also, the additives have led to increasing costs of infant formula despite the lack of proven benefits to normal, full term infants. There is a need for more effective regulation of DHA and ARA additives to infant formula.

## Background

Fatty acids are important components of breast-milk, contributing to the healthy development of children in various ways. Most infant formula manufacturers now add manufactured docosahexaenoic acid and arachidonic acid, commonly known as DHA and ARA. They are manufactured in the sense that they are created through industrial processes, and not created naturally in human breastmilk. Concerns were raised about these additives as early as 1996 [1], but they remain unresolved. New issues are becoming visible as it is recognized that infant formula is becoming a global commodity, one that requires coherent global regulation.

This analysis begins with an overview of the regulatory agencies at national, regional, and global levels. That is followed with a discussion of major problematic issues relating to fatty acids added to infant formula. This then leads to a call for more systematic regulation of infant formula in the U.S. and worldwide.

Most binding regulations relating to infant formula come from national governments, and apply within their jurisdictions. Typically they are administered through the nation’s ministry or department of health. In the U.S., primary responsibility for implementation of the rules relating to infant formula is lodged with the Food and Drug Administration (FDA), in the U.S. Department of Health and Human Services. In large countries, sub-national layers of government may have some authority to issue binding regulations relating to infant formula.

Some regional bodies have the authority to issue binding regulations relating to infant formula, such as Food Standards Australia New Zealand (FSANZ). In Europe, the European Food Safety Authority (EFSA) works on risk assessment, while the European Commission issues Directives and Regulations. The member countries are asked to implement the Directives through their national law. The Common Market of the South, MERCOSUR, in Latin America, works to harmonize its members’ national legislation relating to nutrition, but its recent regulation on nutrition claims excludes infant formula from its coverage [2].

The nations of the world have not given any global agency the authority to issue binding regulations relating to food, so global agencies are limited to issuing recommendations. The major global inter-governmental agency issuing recommendations relating to food is the Codex Alimentarius Commission, organized jointly by the Food and Agriculture Organization of the United Nations (FAO) and the World Health Organization (WHO). Apart from FAO and WHO, several other agencies are concerned with food issues, such as the United Nations Children’s Fund (UNICEF) and the World Food Programme (WFP). However, at the global level, only the Codex Alimentarius Commission issues detailed technical recommendations about how national and regional bodies should regulate in relation to food issues.

The World Trade Organization (WTO) plays an important role in regulating foods in international trade. It normally accepts the recommendations of the Codex Alimentarius Commission with regard to food issues, especially in its dispute settlement procedures. For nations that are interested in trading their food products internationally, this often has the effect of turning Codex recommendations into binding regulations, even though they do not have that formal legal status. The WTO also accepts consensus-based standards from other international organizations, such as the International Organization for Standardization (ISO).

Several non-governmental organizations play important roles relating to the regulation of infant formula. For example, the International Baby Food Action Network, together with its national branches, urges national governments to implement the International Code of Marketing of Breast-milk Substitutes.

There are lobbying groups for the food industry that can influence regulations, including specialized agencies such as the Infant Formula Council based in the U.S., the Infant Nutrition Council based in Australia, and the International Association of Infant Food Manufacturers, based in Switzerland. The major manufacturers of infant formula and of important ingredients and additives also lobby the regulatory agencies directly.

The Switzerland-based International Organization for Standardization and AOAC International “have agreed to cooperate on the development of infant formula and adult nutritional related standards [3].” AOAC is the Association of Analytical Communities.

The U.S. Pharmacopeia Convention (USP) is a non-governmental organization that recommends scientific standards for pharmaceuticals, foods, and other products. Its food-related recommendations are published regularly in its *Food Chemicals Codex*. The FCC was originally launched by the National Academy of Sciences, but transferred over to the USP in 2006. The USP works only with issues such as the identity and the purity of ingredients. It does not consider their functions in terms of effects on health.

There is effort at self-regulation and consumer education by an association of manufacturers of the additives through the non-governmental Global Organization for EPA and DHA Omega-3s [4].

The global standard for infant formula established by the Codex Alimentarius Commission is designated as CODEX STAN 72–1981. It includes a list of required ingredients and names various required quality control measures [5].

Section 3.2 of this standard, on Optional Ingredients, says:

3.2.1 In addition to the compositional requirements listed under 3.1.3, other ingredients may be added in order to provide substances ordinarily found in human milk and to ensure that the formulation is suitable as the sole source of nutrition for the infant or to provide other benefits that are similar to outcomes of populations of breastfed babies.

3.2.2 The suitability for the particular nutritional uses of infants and the safety of these substances shall be scientifically demonstrated. The formula shall contain sufficient amounts of these substances to achieve the intended effect, taking into account levels in human milk.

Brief technical specifications are then provided for a variety of optional ingredients, including DHA.

In this Codex standard on infant formula, DHA is regarded as an optional ingredient, not as a food additive, but some national regulatory agencies describe it as an additive.

Recommendations from the Codex Alimentarius Commission may be adopted by regional bodies and by national governments in various ways. In the U.S., they are codified under U.S. law, and their implementation is overseen primarily by the U.S. Food and Drug Administration. While the FDA has primary responsibility for the composition of infant formula, the Federal Trade Commission (FTC) plays an important role in relation to marketing of the product. The FDA is a regulatory agency, while the FTC is a law enforcement agency. The FTC investigates and assesses advertising claims.

The Federal Food, Drug, and Cosmetic Act defines infant formula in Title 21, Section 321(z) of the United States Code of Federal Regulations. It is “a food that purports to be or is represented for special dietary use solely as a food for infants by reason of its simulation of human milk or its suitability as a complete or partial substitute for human milk (21 U.S. Code 321 (z)).”

Section 350a of the act sets out a list of required nutrients and their minimum and maximum quantities. The list includes protein, fat, essential fatty acids (only linoleate is in the list), fifteen different vitamins, and eleven different minerals. The list conforms to the recommendations of the Codex Alimentarius Commission.

The U.S. Code of Federal Regulations Title 21, Part 106 specifies infant formula quality control procedures. It is mainly about quality control during the manufacturing process, and not directly about the quality of the product that emerges from that process (U.S. Code of Federal Regulations (21CFR106), 2009). Part 107 states the nutrient requirements and other rules regarding labeling, recalls, etc. [6].

There are four major areas in which regulations relating to infant formula can be useful: nutrient content, health claims, safety, and economic value, discussed in the following four sections of this paper. Nutrient claims are about ensuring that the manufacturers make clear and accurate claims about what is in the product. Health claims are about specific anticipated health benefits, and whether use of the product does in fact lead to the health benefits that are claimed. Safety is about whether disease or death is likely to result from using the product, especially in the short term. The concern regarding economic value is about whether the producers make claims that are misleading in order to enhance their economic gain.

The FDA defines “safe” as “a reasonable certainty in the minds of competent scientists that the substance is not harmful under the intended conditions of use”, as specified in the U.S. Code of Federal Regulations at (21 CFR 170.3(i)).

Safety is about harms that would make you worse off than if you had not ingested the substance. Concerns about health impacts usually focus on whether you are better off as a result of ingesting the substance.

Some studies described the absence of anticipated health benefits as a safety issue, but most distinguish the two as different types of quality issues. Failure of infants who use a particular type of infant formula to achieve anticipated weight gains, for example [7], usually would be regarded as a failure to achieve an anticipated health benefit, not as a safety issue.

This study is based on critical analysis of policies and practices described in publicly available documents of the FDA, the manufacturers of fatty acids, and other relevant organizations. The broad framework for this work was set out by the author in his book on *Regulating Infant Formula*, published in 2011 [8].

## Results and discussion

### Claims regarding nutrient content

In the U.S., the Nutrition Labeling and Education Act of 1990, which amended the Food, Drug, and Cosmetic Act, makes a distinction between nutrient content claims and health claims. Nutrient content claims characterize a nutrient in a food. The product should include what it is claimed to include. Health claims characterize a relationship of a nutrient to a disease or health-related condition. Health claims are discussed in the following section.

From time to time the FDA questions manufacturers regarding their nutrient content claims. To illustrate, a warning letter from the FDA listed some of one company’s products and said:

We have concluded that these products are in violation of the Federal Food, Drug, and Cosmetic Act (the Act) and FDA regulations. . . . because their labeling includes unauthorized nutrient content claims. Except for claims regarding percentages of vitamins and minerals for which there is an established Reference Daily Intake, a nutrient content claim cannot be made for a food that is intended for use by infants and children less than two years of age unless the claim is specifically provided for in parts 101, 105, or 107 of the regulations. 21 CFR 101.13(b)(3). . . .

The circumstances under which these claims are permitted are defined in 21 CFR 101.60(c) and 101.54(e), respectively. However, those regulations do not permit these claims for products intended for infants and children under age 2 [9].

The legal status of manufacturers’ claims regarding the DHA and ARA content in infant formula have not been closely examined by the U.S. government. Some of the current claims might violate the law cited in the FDA’s letter.

There may be other nutrient content concerns about infant formula and additives to it, such as whether they are organic or genetically engineered, whether they are kosher, and whether they are halal. There are few well-developed regulations regarding these issues.

Some DHA and ARA additives, made on an industrial scale from fungi or algae, are claimed to be organic while others dispute that claim [10,11]. There has been debate over whether additives such as manufactured DHA and ARA that are made with solvent extraction processes can be classified as organic [12]. Some parties say anything that begins with organic products such as fish, algae, or eggs is organic, while others say taking such products through an industrial process disqualifies them from that category. The FDA made a statement on the issue that did not clarify the matter [13].

### Claims regarding health

#### What evidence for what health claims?

When DHA was first added to infant formula, the pitch to investors was that the product “may help close gaps researchers have found between the development of breast-fed and bottle-fed infants [14].” However, most of the research on DHA and ARA additives focuses on comparing infant formulas with different additives. Little of the research looks into how infant formulas with and without DHA compare with breastfeeding in terms of health effects. It might be that infant formula with DHA produces somewhat better results than infant formula without that additive, but if both produce far worse results than breastfeeding, that would be important to know. Parents need to make well informed choices not only in deciding which infant formula to use but also in deciding whether to use formula rather than breastfeed.

Using newer versions of the additive might have better health effects than older versions, and thus bring the product somewhat closer to the performance of breastfeeding. However, claiming that any infant formula with the fatty acid is “closest to human milk” or “as close as possible to human milk” is not the same as saying it is close to human milk. The claim may be misleading if the difference between “closer to” and “close to” escapes people’s notice.

When the manufacturers make claims about the health effects of their DHA and ARA that are added to infant formula, they pick out studies that support their views, and ignore studies that do not. They do not clearly identify the specific studies that supposedly support their specific claims. There is no clear consensus about what indicators should be used for the major claims.

To illustrate, one manufacturer’s website said:

Certain studies have also shown that infants fed formula supplemented with DHA and ARA exhibited:

• Improved mental development

• Better visual acuity

• Significantly lower blood pressure at age six which may reduce the risk of cardiovascular disease later in life

Preterm infants who were fed DHA and ARA supplemented formula exhibited normal growth in terms of weight, length and head circumference and improved visual and mental development [15].

The manufacturer listed 28 studies said to be “Research Supporting the Importance of DHA & ARA in Infant Development,” with an additional seven listed as “Research Supporting the Importance of DHA for Pre-term and Near-term Infants.” It is not clear which of the studies is supposed to support which of the specific claims. Many were published prior to 2000, and thus have little relevance to current versions of manufactured DHA and ARA. Many of the studies listed have no relevance to any of the claims.

The list did not mention the many studies that found no significant health benefit from fatty acids added to infant formula. It did not mention that several major meta-analyses found no significant effect [16-18].

(The article by Qawasmi and colleagues published online by *Pediatrics* in June 2012 [16], concluded, “LCPUFA supplementation of infant formulas failed to show any significant effect on improving early infant cognition”. It was followed by another online article with a similar title in December 2012, this time concluding, “Current evidence suggests that LCPUFA supplementation of infant formulas improves infants’ visual acuity up to 12 months of age.” The journal published my letter inquiring about the reasons for this change, but there has been no reply. Their second article and my letter are available at http://pediatrics.aappublications.org/content/early/2012/12/12/peds.2012-0517.abstract/reply#pediatrics_el_54993. A third and final version of the article appeared in print in *Pediatrics* in January 2013, described at http://www.ncbi.nlm.nih.gov/pubmed/23248232. Its conclusion was the same as that in the December 2012 version. Apparently, seven studies were added to the set of twelve originally reviewed, leading to the change in the conclusion.)

The manufacturer said only that “certain studies” showed the effects that were claimed, but they did not specify which ones. There are also studies that fail to show those effects. The manufacturer has not demonstrated that the dominant trend in the relevant studies supports their claims.

Another page on the same website discussed “The Benefits of DHA and ARA During Infancy” under various categories: cognition, vision, immune response, etc. For each category, the document said there were studies that supported their claims regarding these benefits. At the end there was a link to an obscure list of references, with no indication of which were supposed to support which claims [19].

The manufacturer said, “Clinical studies have demonstrated numerous benefits for infants receiving DHA and ARA supplemented formula, including improved mental and visual development [20-22],” but it did not say which studies.

The manufacturer did not say which, if any, of the studies it listed were specifically about its currently featured product, trademarked as *life’s DHA*. For advertising purposes the manufacturer apparently wants *life’s DHA* to be viewed as very different from earlier versions of the manufactured fatty acid, but at the same time it suggests that earlier research based on other versions of DHA and the regulations on them should apply just as well to this latest version [23].

There is no consensus as to what indicators would be appropriate for assessing each of the different types of health claims made for fatty acids added to infant formula. Questions have been raised about the Bayley Scales of Infant Development (BSID) used in the meta-analysis by Qawasmi and colleagues, and in many other studies as well [16,24]. The critics argue that these scales, used primarily for children up to 18 months of age, are not appropriate for assessing how these ingredients affect physical and mental development in the long run.

The FDA has expressed its doubts about manufacturers’ health claims:

16. What is the evidence that addition of DHA and ARA to infant formulas is beneficial?

The scientific evidence is mixed. Some studies in infants suggest that including these fatty acids in infant formulas may have positive effects on visual function and neural development over the short term. Other studies in infants do not confirm these benefits. There are no currently available published reports from clinical studies that address whether any long-term beneficial effects exist [25].

#### Which DHA?

When a manufacturer listed “Research Supporting the Importance of DHA & ARA in Infant Development,” it did not say all these studies would show the importance of *manufactured* DHA and ARA. The manufacturer implicitly assumed that evidence relating to human DHA and ARA as found in breast-milk would apply just as well to their manufactured versions. However, the manufacturer’s claims were specifically about “formula supplemented with DHA and ARA,” meaning the manufactured versions.

DHA in breast-milk tested in a chemistry laboratory might appear to be similar to a manufactured DHA. However, the real test of equivalence is whether they function in the same way. Do they lead to the same results for infants on relevant health indicators? The health effect of the two should not be claimed to be the same unless their performance is demonstrated to be the same [26].

It is also important to distinguish among various types of manufactured DHA. U.S. Pharmacopeia’s Food Chemicals Codex distinguish three types of algae-based DHA, corresponding to three different algal oils: schizochytrium, crypthecodinium, and ulkenia.

There are other DHAs that are not in the Food Chemicals Codex. For example, there is an egg-based DHA:

Baby’s Only Essentials® DHA & ARA Fatty Acid is naturally derived from the goodness of Egg Phospholipids using an aqueous (water) process. This differs from C. cohnii oil (algae) & M. alpina oil (fungus) used in all other organic and conventional infant formulas, which are treated with hexane solvent, acid, and bleach [27].

It is not clear how the different types of manufactured DHA compare with regard to their safety or their effectiveness. If the newer ones are said to be improvements, that implicitly acknowledges that the earlier ones must have been deficient.

#### Regulations regarding health claims

U.S. rules regarding health claims have been summarized as follows:

. . . . the Nutrition Labeling and Education Act . . . . set a high threshold for the scientific standard under which the U.S. Food and Drug Administration (FDA) may authorize health claims, this standard is known as the significant scientific agreement (SSA) standard. Subsequent legislation known as the Food and Drug Administration Modernization Act (FDAMA) provided an alternative to FDA review of the health claim where an U.S. government scientific body other than FDA concluded that there is SSA for a substance/disease relationship ([28], also see [29]).

Comparable regulations of health claims came into force for the European Union in January 2007 [30].

Following the practice in a number of other countries, in 2001 the FDA accepted the addition of manufactured DHA to infant formulas. However, the agency has not affirmed that this addition is beneficial, and it has not explicitly authorized the manufacturers to make claims regarding their benefits.

The FDA explains its role as follows:

4. Does FDA approve infant formulas before they are marketed?

No, FDA does not approve infant formulas before they can be marketed. However, all formulas marketed in the United States must meet federal nutrient requirements and infant formula manufacturers must notify the FDA prior to marketing a new formula. If an infant formula manufacturer does not provide the elements and assurances required in the notification for a new or reformulated infant formula, the formula is defined as adulterated under Section 412(a)(1) of the FFDCA and FDA has the authority to take compliance action if the new infant formula is marketed ([25], also see [31]).

From time to time FDA responds to petitions regarding health claims. There is a good illustration of how such petitions are handled in FDA’s response to a petition regarding a health claim for whey-protein partially hydrolyzed infant formula [32].

The FDA has not endorsed the manufacturers’ claims regarding the health benefits of adding DHA to infant formula. It *may* authorize health claims, under the standard known as significant scientific agreement (SSA). The law does not say the FDA *must* authorize health claims. There is no published SSA for DHA in infant formula [33].

The Infant Formula Council, a lobbying group for the manufacturers, said, “The use of LCPUFAs [long chain polyunsaturated fatty acids] in infant formulas has been reviewed and supported by the U.S. Food and Drug Administration [34].” Given FDA’s explanation that it does not approve infant formula and its statement that the evidence regarding the benefits of DHA and ARA in infant formula is mixed, it is difficult to guess what the Council might have meant by “reviewed and supported.”

There is little effective regulation with regard to health claims or the general nutritional adequacy of infant formula and its additives. The regulators regularly accept the manufacturers’ claims that the currently proposed versions are superior, but do not explore how the previous ones might have been deficient.

Current regulations for infant formula focus on ensuring that particular components are supplied in specified quantities, based on an ingredients list from the 1980s. They imply that any formula that includes the specified ingredients in the required amounts is safe and nutritionally adequate. However, breast-milk is a complex, changing, living thing, and not simply a static collection of inert ingredients. Many ingredients in breast-milk are absent in infant formula, or are provided in infant formula in forms that are different from those in breast-milk. There is little recognition of the fact that ingredients can act differently in different contexts.

It should not be assumed that DHA additives that are manufactured by one process have the same health effects as DHA manufactured by another process. Similarly, it should not be assumed that the health effects of manufactured DHA in infant formula are the same as the health effects of natural DHA in breast-milk. These propositions need to be verified empirically.

### Claims regarding safety

The FDA’s treatment of additives is different from its approach to food in general. Its position is that “companies that want to add new additives to food bear the responsibility of providing FDA with information demonstrating that the additives are safe. FDA experts review the results of appropriate tests done by companies to ensure that the additive is safe for its intended use. . . . Certain food ingredients, such as those with a long history of safe use in food, do not require premarket approval [31].”

#### GRAS determination

The FDA’s *Guidelines Concerning Notification and Testing of Infant Formulas* say:

A manufacturer must notify FDA 90 days before the first processing of any infant formula for commercial or charitable distribution for human consumption that differs fundamentally in processing or in composition from any previous formulation produced by the manufacturer [35].

These incremental changes are handled by determining whether the proposed additives are GRAS, Generally Regarded as Safe. It is not the FDA itself that assesses their safety. The manufacturer is supposed to assess their safety and notify the FDA of its findings. To illustrate, in 2001 the FDA responded to requests from manufacturers to have their versions of these fatty acids, ARASCO and DHASCO, characterized as GRAS. In its response, the FDA said:

The agency has not, however, made its own determination regarding the GRAS status of the subject use of ARASCO and DHASCO [36].

The FDA had no questions about the documents submitted to it about the companies’ requests for GRAS status for these products, and it acknowledged that the FDA had not made its own independent determination regarding their status. The FDA did not say whether it agreed with the requests.

The FDA’s Inventory of GRAS Notices shows that the agency records the details of the notifications regarding GRAS that it receives [37]. The FDA does not affirm that the product is safe.

The GRAS determination process is explained in FDA’s *Frequently Asked Questions about GRAS*[38]. The answer to question 10 is of particular interest:

Does FDA currently have a program to affirm that one or more uses of a food substance are GRAS?

In a proposed rule that FDA published in 1997 (62 Fed. Reg. 18938; April 17, 1997), FDA explained why the agency could no longer devote resources to the voluntary GRAS affirmation petition process that is described in 21 CFR 170.35(c) and proposed to abolish that process and replace it with a notification procedure. The agency has not yet issued a final rule however, and the petition procedure remains in the agency’s regulations. However, at this time FDA is not committing resources to the review of GRAS affirmation petitions.

In other words, the FDA does not evaluate the GRAS petitions it receives.

The question is not whether these optional ingredients are in fact safe to use. The GRAS question is about whether they can reasonably be *assumed* to be safe. The distinction is important. To illustrate, one could be unsure whether infant formula made with genetically modified soy is safe for infants, but at the same time be certain that it should not be *assumed* to be safe.

The GRAS concept was developed to avoid having to scientifically assess familiar products that were known to be safe on the basis of extensive experience in diverse conditions. In the case of novel products such as manufactured DHA and ARA intended for highly vulnerable infants, the designation seems unreasonable. Moreover, depending on the manufacturers of the products to make the judgment about whether the product should be considered GRAS seems unreasonable. Children should not be made to bear the risk entailed in making that assumption.

#### Postmarket surveillance

The FDA’s acceptance of the GRAS notification allows manufacturers to begin marketing the product. However, in its letters to the manufacturers, the FDA said:

FDA would expect any infant formula manufacturer who lawfully markets infant formula containing ARASCO and DHASCO to monitor, through scientific studies and rigorous post-market surveillance, infants who consume such a formula. We also would expect regular reports of such studies and post-market surveillance. Because the use of ARASCO and DHASCO in infant formula would be based on the GRAS provision of the FFDCA, we also would expect that these reports would not be considered to be confidential so that the broader scientific community can contribute to this continuing evaluation [36,39].

Thus, there was an expectation of surveillance of the products’ safety and health effects after marketing was underway.

In response to an inquiry from the Cornucopia Institute in 2009, the FDA said it had received no postmarket surveillance reports on the safety and effectiveness of these products. Nevertheless, the industry proceeded with large-scale advertising campaigns promoting infant formula and many other food products based on its claims about the benefits of these additives [40]. There is no indication that the manufacturer has ever done such studies [10,41].

Numerous “Adverse Reaction Reports” that appear to be related to DHA/ARA in infant formula have been submitted to the FDA. The Cornucopia Institute has made some of them available on its website [42]. The adverse reactions described by mothers included gas, diarrhea, vomiting, bowel obstruction, gastric reflux, colic, and constipation.

It is unfortunate that FDA relies on the industry for post-market surveillance of the effects of optional ingredients because the value of the industry’s reports would be highly questionable. It is even more problematic that the FDA now seems to be indifferent to the failure of the industry to conduct such studies.

### Concerns relating to economic values

Safety refers to possible harm to health, but there is also the possibility of economic harm. The risk is high, given the strong economic motivations of those who promote infant formula and additives to it. It appears that several infant formula manufacturers add various ingredients, make dubious claims about their health benefits, and increase their products’ prices in order to draw economic benefits from those claims. Several manufacturers put out specialized infant formulas with prices that are considerably higher than the generic “store brands”. There might be some truth to the health claims, but reasons to doubt whether they are sufficient to justify their increased economic costs.

This pattern is evident in relation to the addition of DHA and ARA to infant formula. In the 1990s, a stock promotion said:

Infant formula is currently a commodity market, with all products being almost identical and marketers competing intensely to differentiate their product. Even if Formulaid had no benefit, we think that it would be widely incorporated into most formulas, as a marketing tool and to allow companies to promote their formula as ‘closest to human milk’ [43].

The infant formula business is lucrative because the profits per unit are high, and also because huge growth is anticipated, especially in Asia. Additives tend to increase the already high profit margins.

Since the economic incentives are so strong, new additives may be promoted without sufficient regard for their effect on infants’ health. Some additives appear to be designed to exploit parents’ willingness to pay higher prices to gain every possible benefit for their children.

One might argue that the increased cost associated with the addition of fatty acids to infant formula is not large. But that cost adds up quickly for private or public agencies that purchase large quantities of the product. The biggest purchaser is the United States’ Special Supplemental Program for Women, Infants and Nutrition, commonly known as WIC. That program supplies about half the infant formula used in the U.S., at no cost to the families. According to the nongovernmental Center on Budget & Policy Priorities, “WIC appears to be spending more than $90 million extra annually—or more than 10 percent of its total spending on infant formula—to provide formulas with ingredients that neither USDA nor the FDA has assessed with regard to their benefits [44,45].”

Despite its responsibilities as an agency of the U.S. government, WIC does not have the power to prevent this. As explained by Maureen Minchin:

A 2004 legislative change removed WIC’s ability to determine which of the formulas it wanted from a tendering company; companies could determine what formula they offered to WIC, at what price. If they offered the expensive or novel brands, WIC had no choice but to become the inadvertent marketer of such products, despite the lack of scientific proof of either safety or efficacy, or the preferences of the WIC authority. Attempts as late as 2010 to study the new ingredients were stymied by high-powered industry lobbyists . . . [46].

This has major consequences because many women both in and out of the WIC program view WIC’s providing particular types of infant formula as indicating government endorsement of them.

In the U.S., the Federal Trade Commission has primary responsibility for issuing cautions and regulations relating to commerce. In 2010 it sent letters to eleven companies that promote fatty acid supplements in infant food, asking them to “review their product packaging and labeling to make sure they do not violate federal law by making baseless claims about how the supplements benefit children’s brain and vision function and development [47].”

A few months later, the FTC issued an order settling charges that certain manufacturers had made false and unsupported claims that their children’s multivitamins contained a significant amount of DHA and promoted healthy brain and eye development. The companies agreed to pay $2.1 million to provide refunds to consumers [48]. Perhaps the FTC should examine questionable claims related to fatty acids in infant formula.

## Conclusions

Infant formula is sometimes regarded as a standardized commodity, with any product that meets the legal specifications presumed to be as good as any other. When the basic recipe was worked out in the 1980s, the argument was that it would be fully adequate for all normal children. However, product differentiation by brands, advertising, and the stream of various optional ingredients and additives imply that some versions of infant formula are superior. This means others are inferior. This raises serious questions about whether the basic recipe for infant formula really is nutritionally adequate.

It is impossible for parents to scientifically judge the validity of the various health claims regarding infant formula. No independent agency compares different infant formula products. If an additive can be clearly shown to be beneficial for infants in the general population, without simultaneously resulting in significant harm, it should be added to the basic list of required ingredients for infant formula. Rather than regard such ingredients as optional and possibly available only to high-income families, all formula-fed infants should get them. Good research would have to be done to determine that a proposed new component should be required in all infant formula.

Exceptions could be made for infants with particular needs. DHA and ARA additives might be appropriate for some infants, but not for the great majority of them. Formulas for infants with particular needs could be defined as pharmaceuticals, and distributed on a prescription basis, rather than through commercial marketing.

Another option would be to categorize some of the optional ingredients and additives as *medical foods*. According to the FDA,

The term medical food, as defined in Section 5(b) of the Orphan Drug Act (21 U.S.C. 360ee (b) (3)) is “a food which is formulated to be consumed or administered enterally under the supervision of a physician and which is intended for the specific dietary management of a disease or condition for which distinctive nutritional requirements, based on recognized scientific principles, are established by medical evaluation [49].”

This approach would encourage more thorough governmental monitoring of optional ingredients and additives.

Many of the issues raised here regarding fatty acids in infant formula may be relevant to other additives, such as prebiotics and probiotics [50-53], nucleotides [54], and sweeteners [55]. Some additives might have unproven benefits, be unnecessarily costly, or expose infants to new kinds of harms. Comparable issues arise not only for optional ingredients and additives, but also for other types of infant formula, such as soy-based infant formula.

Overall, the major finding here is that in the U.S. the quality of infant formula and related optional ingredients and additives are not adequately regulated to ensure that the interests of children and their families are protected. Infants are exposed to potential risks that have not been adequately assessed. Also, the additives lead to increasing costs of infant formula despite the lack of proven benefits to normal, full term infants.

Contrary to many people’s assumptions and expectations:

1. The U.S. Food and Drug Administration does not systematically assess health claims related to infant formula or to specific additives such as fatty acids.

2. The U.S. Food and Drug Administration does not systematically assess the safety of infant formula or to specific additives such as fatty acids.

3. The U.S. Food and Drug Administration does not systematically evaluate the manufacturers’ claims regarding safety and health for infant formula or specific additives such as fatty acids.

4. There is no independent systematic field research on health claims or safety related to infant formula or specific additives such as fatty acids.

This pattern raises questions about whether government agencies in the U.S. are carrying out their obligations to ensure the safety and nutritional adequacy of infant formula in its various forms. The same questions should be raised for every country.

The major infant formula producers operate globally, and they are planning a massive push into Asia and other parts of the world. One consultancy firm says, “In 2011 the infant formula market is still growing rapidly, with the development of markets like Asia, particularly China, with a growth rate close to 20 % per year, Eastern Europe, and in a lesser extent Middle East and Latin America [56].”

Another report says China’s baby food and drink market is expected to be worth more than $15 billion by 2015, with perhaps 80 percent of this comprised of infant formula [57].

In this rapid globalization of the infant formula market, the fatty acids are going along, as indicated by one manufacturer’s sole-source supply agreement with Fonterra, the world’s largest dairy exporter [58].

With the infant formula industry having enormous global reach, the model of regulation under separate national jurisdictions is outdated. The nations of the world should act together to strengthen quality control for infant formula and other foods intended for children. There is a need for a global perspective in regulation, one that is based on appreciation of differences in local circumstances.

## Abbreviations

AOAC: Association of Analytical Communities; ARA: Arachidonic acid; BSID: Bayley Scales of Infant Development; CFR: U.S. Code of Federal Regulations; DHA: Docosahexaenoic acid; EFSA: European Food Safety Authority; FCC: Food Chemicals Codex; FDA: U.S. Food and Drug Administration; FAO: Food and Agriculture Organization of the United Nations; FDAMA: U.S. Food and Drug Administration Modernization Act; FSANZ: Food Standards Australia New Zealand; FTC: U.S. Federal Trade Commission; GRAS: Generally Regarded as Safe; ISO: International Organization for Standardization; MERCOSUR: Common Market of the South; SSA: Significant Scientific Agreement; UNICEF: United Nations Children’s Fund; USP: U.S. Pharmacopeia Convention; WFP: World Food Programme; WHO: World Health Organization; WTO: World Trade Organization.

## Competing interests

The author declares that he has no competing interest.

## Authors’ information

George Kent is Professor Emeritus with the University of Hawai’i. He was a professor in the Department of Political Science from 1970 until his retirement in 2010.

He continues to teach an online course on food policy issues as a part-time faculty member with the Centre for Peace and Conflict Studies at the University of Sydney in Australia and also with the Social Transformation Concentration at Saybrook University in San Francisco.
